# Multistage Background Field Removal (MUBAFIRE)—Compensating for *B*_0_ Distortions at Ultra-High Field

**DOI:** 10.1371/journal.pone.0138325

**Published:** 2015-09-22

**Authors:** Johannes Lindemeyer, Ana-Maria Oros-Peusquens, N. Jon Shah

**Affiliations:** 1 Institute of Neuroscience and Medicine 4, INM-4: Medical Imaging Physics, Forschungszentrum Jülich, Jülich, Germany; 2 Department of Neurology, Faculty of Medicine, JARA, RWTH Aachen University, Aachen, Germany; University Hospital of Würzburg, GERMANY

## Abstract

The investigation of tissue magnetic susceptibility and the resultant magnetic field offers a new avenue for quantitative tissue characterisation by MRI. One crucial step in mining the phase and field data for relevant tissue information is the correction of externally induced field shifts. This article outlines a multistep approach comprising several methodologies for background field removal. The virtues of *B*_0_ long-range variation detection and compensation of more localised external disturbances are unified in a sequential filter chain. The algorithm is tested by means of a numerical Monte Carlo simulation model and applied to *in vivo* measurements at 3T and 9.4T as well as to a fixed brain tissue measurement at 9.4T. Further, a comparison to conventional filter types has been undertaken.

## Introduction

The data acquired in MRI measurements are complex-valued and thus characterised by a real and imaginary part, or, equivalently, by magnitude and phase. In most clinical applications, however, only the magnitude information is used and the phase information is typically discarded. Besides advanced imaging techniques that utilise phase for contrast enhancement or contrast generation (such as MR angiography or flow imaging), the relation between phase and the static magnetic field and its usefulness for the characterisation of pathology has attracted increasing attention since the late nineties (e.g. [1, 2]). The potential of phase information is only beginning to gain recognition in clinical research via applications such as susceptibility weighted imaging (SWI, [3]) and more recently quantitative susceptibility mapping (QSM, [4]). Several strategies for the reconstruction of tissue magnetic susceptibility based on phase data have been introduced recently (e.g. [4–9]). Both local phase and local magnetic susceptibility distributions have been shown to exhibit excellent tissue contrast in the human brain based on its microscopic structure (e.g. [10, 11]). Unfortunately, despite the remarkable quality of the phase contrast induced by local structures, as reported at high field strengths (e.g. [6]), macroscopic field distortions are roughly an order of magnitude stronger and hence prevent a direct inspection of phase information relevant to brain structure. These distortions originate from a number of different sources. First, the static field (*B*_0_) is to some extent inhomogeneous at the *ppm* level with a smooth, slowly varying behaviour. Furthermore, the intrinsic inhomogeneous magnetic susceptibility distributions in the human head and body as well as those caused by the RF coil and the patient table generate medium to long range field distortions relevant to *in vivo* measurements. Additionally, in the head in the vicinity of air, bone and soft tissue boundaries large susceptibility variations are encountered. These can induce large field changes which cannot be fully removed by the commercially available shim systems ([12–14] or [15], Chpt. 6).

In pursuit of the aim of investigating local phase and susceptibility contrast, the field distortions originating from outside the volume-of-interest (VOI) need to be removed whilst preserving local contrast. Background field removal (BFR) has been approached using numerous strategies. Thus far, the most prominent examples are high-pass or Gaussian filters [16], polynomial filters [5], filters utilising dipole or susceptibility distributions [17, 18] and, finally, filters based on the harmonic nature of background field distortions, such as SHARP [19, 20]. Whilst filters based on dipoles or susceptibility distributions are the optimal choice when fitting field variations originating in the brain geometry and in local disturbances, such as blood vessels or air-filled cavities, harmonic filters perform more accurately in removing smooth and long-range field variations.

In an earlier work, the *SPherical Harmonic INhomogeneity-suppresing eXpansion* (SPHINX) algorithm for the identification and eradication of harmonic field contributions was introduced [21]. Here, we present a stepwise BFR algorithm addressing the various sources of distortions one by one in separate, sequential steps [22–24] including SPHINX as one central filter component. This algorithm, named *MUltistage BAckground FIeld REmoval* (MUBAFIRE), is characterised in detail. Due to the inclusion of complementary strategies, it is superior to existing, standalone filters. These benefits are illustrated on simulated data and its applicability is tested on data from investigations being carried out at our Institute on human scanners at fields of 3T and 9.4T.

## Materials and Methods

### Ethics Statement

The *in vivo* measurements presented herein were performed on healthy volunteers; written informed consent was obtained prior to the measurements. The 3T study as well as the 9.4T measurement were performed in agreement with the Ethics Committee of the Medical Faculty of the Rheinisch-Westfälische Technische Hochschule Aachen (RWTH Aachen University, University Hospital, Aachen, Germany). The trial was conducted according to the declaration of Helsinki (6th revision, 2008). For the *post mortem* application, we used a fixed female brain obtained from the brain donor programme of the Heinrich-Heine-Universität Düsseldorf (University of Düsseldorf, Düsseldorf, Germany) scanned in a custom-made cylindrical acrylic glass container filled with formalin. All data were processed anonymously.

### Theory

The phase, *φ*, of the MR signal observed in a gradient echo measurement reflects deviations of the local field, *b*, in a voxel from the main field, *B*_0_. The phase accumulated over time after excitation generally exhibits a linear relationship with the local field shift *φ* = *γ* ⋅ *b* ⋅ *t*_TE_. Non-linear contributions will be ignored in the following. An unwrapping algorithm, such as PRELUDE [25], can be used to correct for phase aliasing and a field map is generated from the phase information utilising single or multiple echo acquisitions (e.g. [26]). Inhomogeneities of *B*_0_ (also present in the absence of the sample) and field distortions induced by sources outside the volume of interest (e.g. from the body to the brain) will be referred to as ‘field distortions’, *b*_dist_, while field shifts due to the inner structure of the object of interest will be called ‘internal field’, *b*_int_. Thus,

$$b=b_{\text{dist}}+b_{\text{int}}.$$ (1)

The removal of *b*_dist_ can be facilitated by applying judiciously chosen filters as presented below.

#### Gaussian

The *Gaussian Filter* (GF) attempts this by convoluting *b* with a Gaussian kernel with standard deviation, *σ*, of several voxels width. Having the effect of a high-pass filter, the difference, *b*_int_ = *b* − *b*_dist_ ≈ *b* − [*b* * exp(−*r* ^2^/2*σ* ^2^)], enhances the contribution from local variations with high spatial frequency (* symbolises three-dimensional convolution and *r* is the radial distance). This is also known as *homodyne filtering* (see [27], p. 552 or [28]) in the context of SWI post-processing (e.g. [5]). We use an explicit convolution with limited kernel size that excludes non-mask voxels in the weighting process for each individual voxel to avoid edge effects [29]. Gaussian filters aggressively reduce contrast as they do not take the origin of field variations into account. This is unacceptable for quantitative purposes.

#### Polynomials

Although three-dimensional polynomials are a common fitting approach for background fields, we will consider only the 1^*st*^ order (constant and linear gradients). Higher orders of the polynomial filter (POLF) do not, in general, follow a certain physical solution of the static field problem.

#### Dipole Filtering

Salomir et al. [30] as well as Marques and Bowtell [31] showed that distortions of the static magnetic field originating in susceptibility differences can be approximated by convoluting the underlying distribution of magnetic susceptibility, *χ*, with the field of a magnetic dipole, *d* = (3 cos^2^ *ϑ* − 1)/(4*π* ⋅ *r* ^3^) (where *ϑ* is the angle towards the direction of *B*_0_). With this assumption, Eq (1) can be expressed as:

$$b_{\text{dist}}=B_{0}\cdot \left(\chi *\;d\right)=B_{0}\cdot [(\chi_{\text{ext}}*\;d)+(\chi_{\text{int}}*\;d)]$$ (2)

(*χ*_int_, *χ*_ext_ ≪ 1, and for the brain mask, *m*: $\chi_{\text{ext}}\left(\vec{r}\in m\right)=0$ and $\chi_{\text{int}}\left(\vec{r}\notin m\right)=0$). De Rochefort et al. [4] presented a minimisation-based reconstruction algorithm to determine the susceptibility distribution based on the induced field shifts. With the same approach, an external susceptibility distribution, *χ*_ext_, responsible for the internal distortions, *b*_dist_, can be estimated using a conjugate gradients solver as described in [32] applied to:

$$\min_{\chi_{\text{ext}}}\|W_{\text{mask}}[b-B_{0}\left(\chi_{\text{ext}}*\;d\right)]{\|}_{2}^{2}+\lambda {\left\|W_{T}\cdot \chi \right\|}_{2}^{2},$$ (3)

(*W*_mask_ = *m* is the weighting matrix, restricting field evaluation to the brain mask; *W*_T_ = *m* and *λ* are the Tikhonov weighting matrix and regularisation parameter). The background field, *b*_dist_ ≈ *B*_0_ ⋅ (*χ*_ext_*d), determined by this *Dipole Filter* (DIPF, also known in the literature as *projection on dipole fields*, PDF [18]), describes the field distortions on a physical basis. Nevertheless, the exclusion of arbitrary externally induced shifts with this method would require the computed volume to be large enough to contain all potential sources, leading to high demands on computing time. There has been recent effort to improve the DIPF computing speed, e.g. by reducing fold-in artefacts in finite volumes [33] or with closed-form solutions replacing the iterative solver algorithms [34].

#### Spherical Harmonics

The novel SPHINX Filter [21] is based on the same physical concept as *B*_0_ shimming (e.g. [35]) and utilises a basis built from spherical harmonics to describe the field generated by outside sources, since these are solutions of the Laplace equation (e.g. [36], p. 117ff). The real regular solid spherical harmonic (SSH) functions, **v**_*ln*_ = *N*_*ln*_ ⋅ *r* ^*l*^ ⋅ *Y*_*ln*_ (for an explanation see Appendix), are naturally orthonormal. Yet, for a non-spherical finite domain, such as a brain mask, their orthonormality is destroyed, so their discrete representation has to be orthonormalised using an iterative Gram-Schmidt-based procedure as shown in the Appendix, Eqs 9–11.

Projection onto the orthonormalised functions, ${\hat{u}}_{lm}$, allows for the formulation of an SSH-approximated expansion of the static field:

$$b_{\text{dist}}\approx \sum_{l=1}^{n}\sum_{m=-l}^{l}c_{lm}\cdot {\hat{u}}_{lm}.$$ (4)

The coefficients are determined by projecting the field, *b*, inside the brain mask onto ${\hat{u}}_{lm}$: $c_{lm}=\langle b|{\hat{u}}_{lm}\rangle$. SPHINX is well suited to approximate smooth field variations with low harmonic order, such as those due to imperfections of the magnet and shimming system. However, the computation of higher order, orthonormalised SSHs is very time consuming. The filter is thus not suited for local perturbations.

#### Multistage Filtering

**Fig 1:**
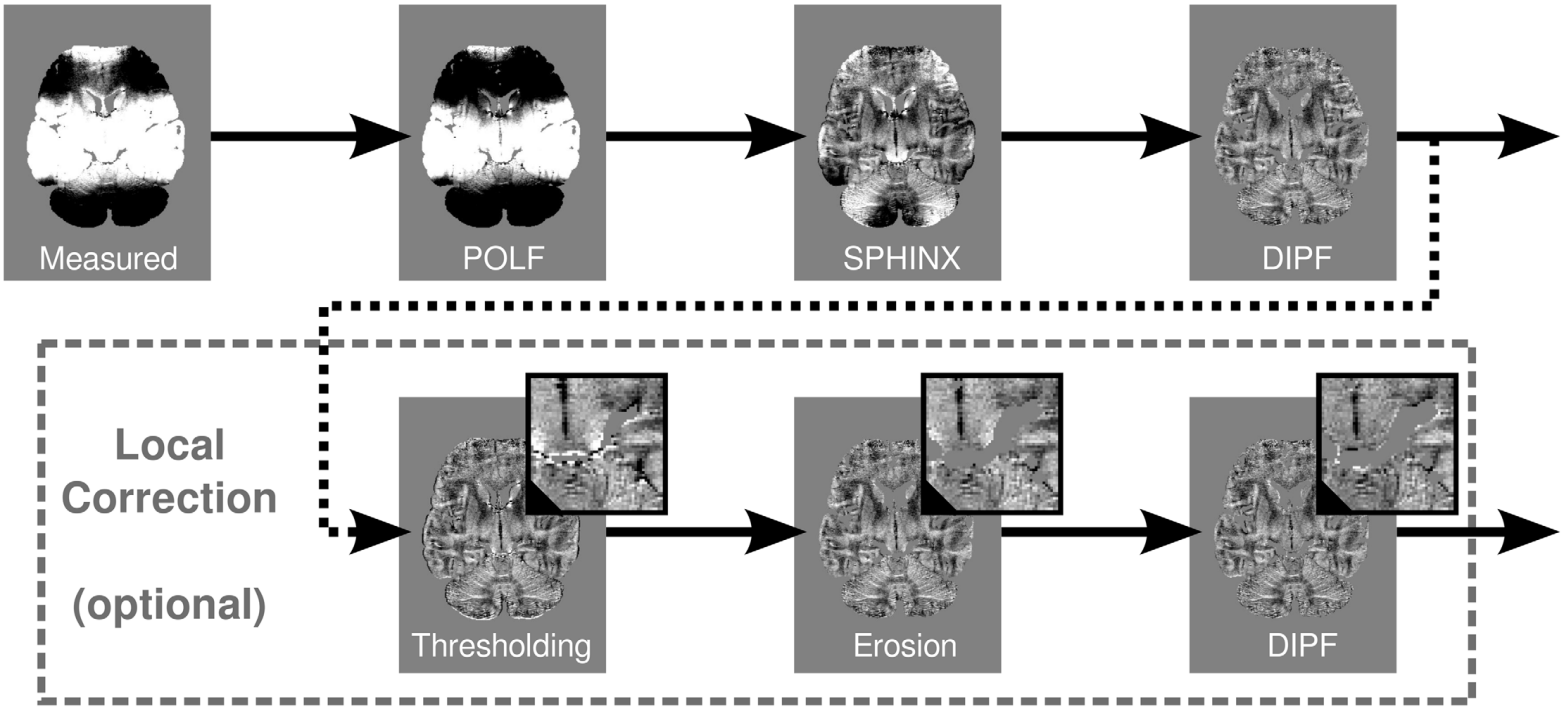
Workflow of the MUBAFIRE algorithm. The raw data are corrected with respect to constant offsets and linear gradients. Then, SPHINX and finally the DIPF are applied. The lower row shows the optional local distortion correction featured by MUBAFIRE Local: After thresholding, a local erosion filter is applied, followed by a final DIPF correcting for local distortions (from [24]).

The novel MUBAFIRE filter chain combines the DIPF, SPHINX and a low order POLF in a hybrid approach. Filters with different optimal domains of applicability are combined such that the disadvantages of a given filter are, to a large extent, covered by the advantages of other filtering steps. The workflow of MUBAFIRE is shown in Fig 1. POLF is applied at 1^*st*^ order (utilising *polyfitn* [37]) to correct for linear gradients and constant shifts of the static magnetic field. Harmonic variations are addressed by SPHINX. In practice, an order of four is employed for fitting. In particular, the correction with POLF and SPHINX includes smooth field variations that are potentially missed by an isolated DIPF correction. Remaining external sources of long- and short-range dipole fields are determined by the DIPF. The corresponding pseudo-susceptibility distribution, *χ*_ext_, being located outside the volume of interest (VOI), is estimated by minimisation as in Eq 3. The field generated by external sources is then subtracted from the map produced by the previous step.

**Fig 2:**
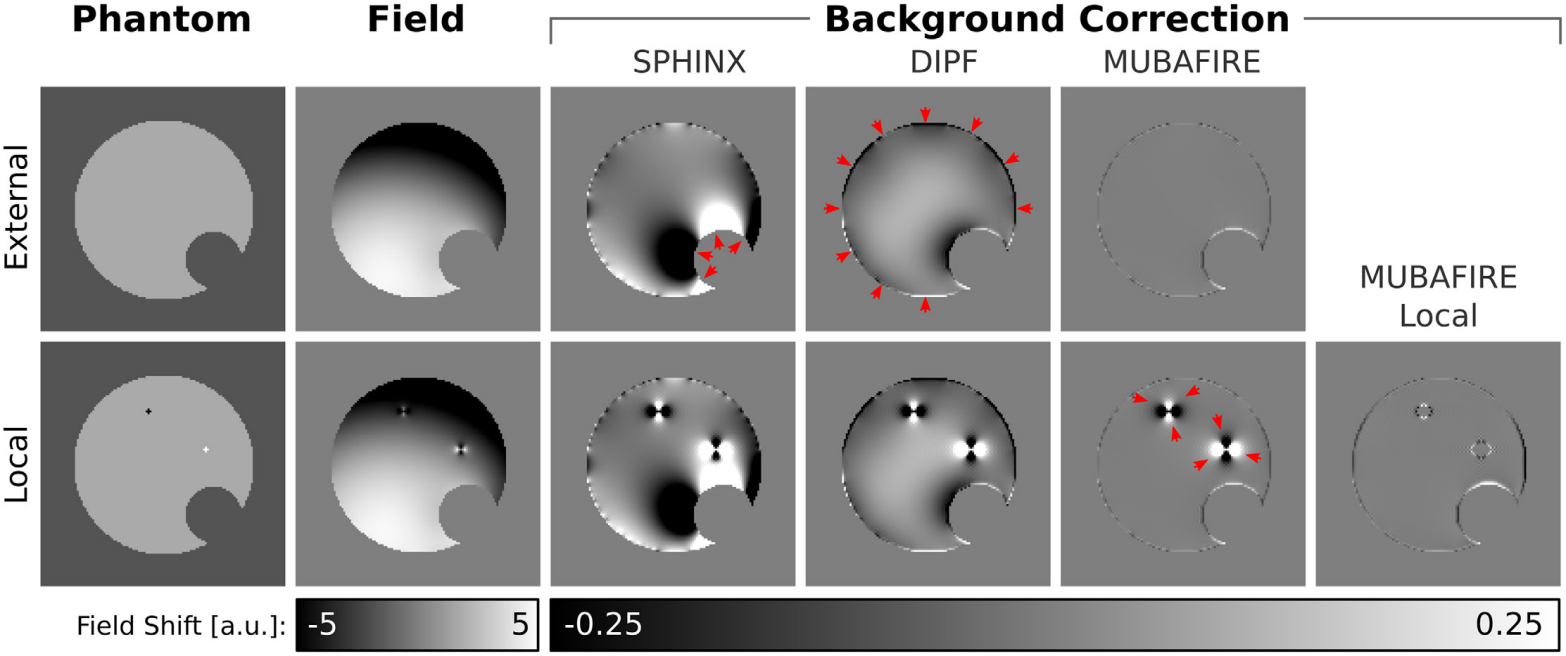
Background Filter Performance. Field shifts are calculated based on the numerical susceptibility phantoms (first column) by dipole convolution and are falsified by artificial harmonic background inhomogeneities (second column). The top row shows a homogeneous phantom, the bottom row includes strong susceptibility spikes. SPHINX, DIPF and MUBAFIRE (Local) are applied to the cases. Red arrows indicate artefacts.

The first row of Fig 2 illustrates the conceptual shortcomings of standalone SPHINX and DIPF and the benefit of uniting the individual filter advantages within MUBAFIRE. The sample field map is calculated by superimposing low order spherical harmonic fields with a dipole convolution of the displayed numerical susceptibility phantom. SPHINX cannot correct for local distortions. In turn, DIPF fails to entirely remove the harmonic inhomogeneity. Neglecting minor edge effects, only MUBAFIRE is able to achieve a homogeneous correction.

**Fig 3:**
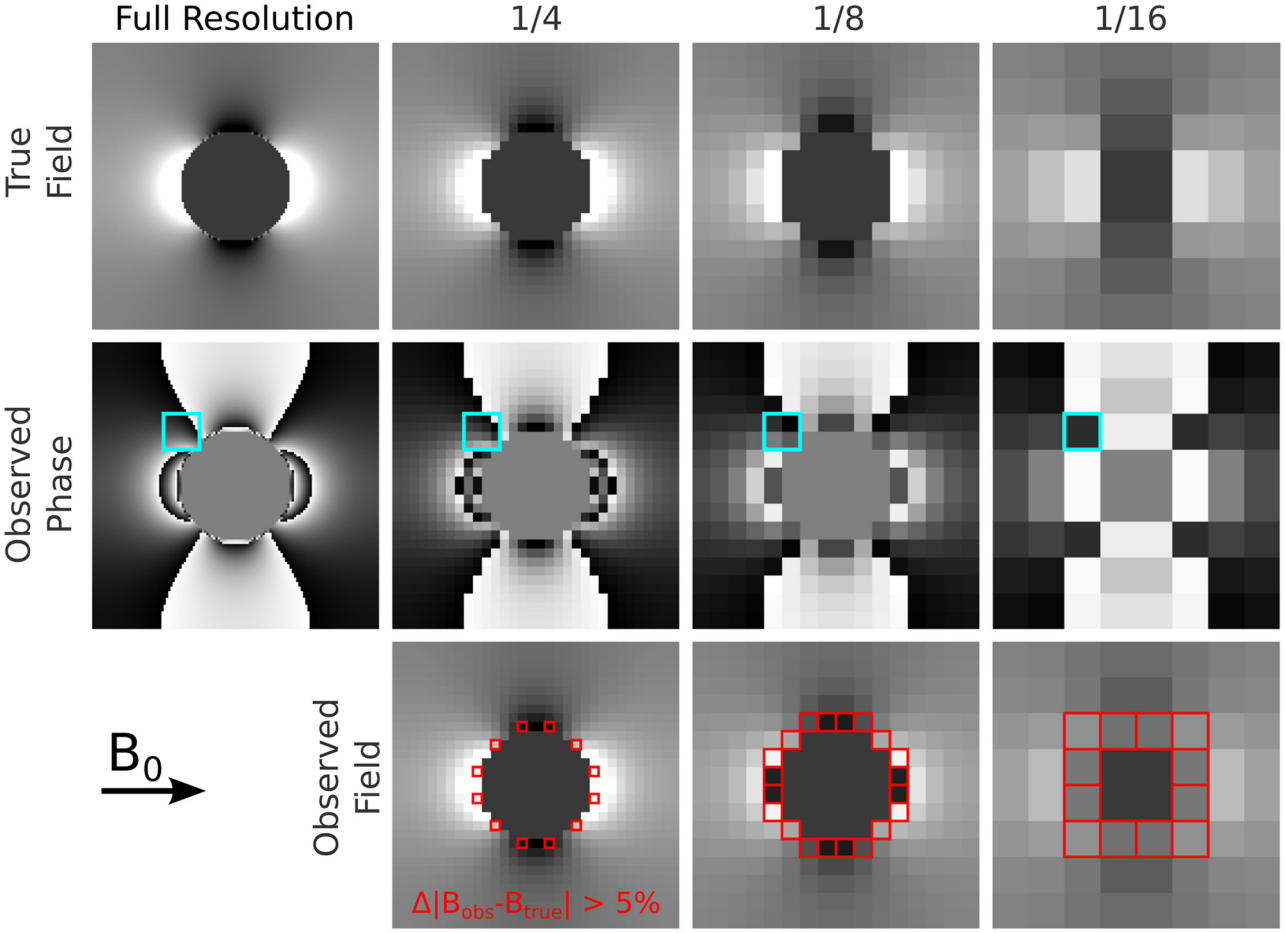
Intra-voxel field gradients. A simulated numerical phantom of a solid sphere with high susceptibility (top left) is resampled in complex domain by 1/4, 1/8 and 1/16 of the native resolution. The field is determined from the phase of the resampled complex signal by unwrapping. Differences higher than 5% between true and calculated field are indicated red. Especially near objects with an extent of only few voxels, but of high susceptibility contrast, phase gradients are strong and lead to incorrect field estimates (right column).

Strong and very localised intrinsic field variations can be generated by blood vessels, small cavities (especially air bubbles in the case of *post mortem* tissue) or erroneous phase/field values that were not excluded during data preparation. Such outliers and their direct surroundings tend to be assigned wrong phase information, caused by rapid field changes on the length scale of a single voxel and by the inability of unwrapping algorithms to describe such effects. This is illustrated in Fig 3. Since conventional QSM lacks appropriate modelling of such field distortions, affected voxels become potential sources for artefacts as they can hardly be excluded during susceptibility reconstruction. Hence, a local field filter, MUBAFIRE Local, was designed. It consists of three additional substeps: thresholding, connecting/erosion and an additional DIPF (see Fig 1, second row). The thresholding step creates a mask based on the field map, $\tilde{b}$, generated by the previous filtering steps and excludes field shifts larger than a certain expected range, $m_{\xi }=\left(\right|\tilde{b}\left|\leq \xi \right)$. The threshold is manually chosen taking into account the nature of the signal and the noise behaviour of the field map, such that only voxels containing erroneous data that would otherwise hinder proper processing of the entire map are removed. Setting *ξ* to a multiple of the standard deviation of the field map inside the mask,

$$\xi =n_{\sigma }\cdot \sigma_{\tilde{b}},$$ (5)

is a beneficial measure, as it reflects the width of the field distribution. Typical values of *n*_*σ*_ (defining the multiple of $\sigma_{\tilde{b}}$ to be used for thresholding) are in the range of 5 to 15.

A customised erosion operation is then used to connect standalone voxels in *m*_*ξ*_, that do not share a common surface, but are arranged as ‘diagonal neighbours’, such as the indexes [*i*, *j*, *k*] and [*i* + 1, *j* + 1, *k*]. The algorithm will in this case additionally exclude [*i* + 1, *j*, *k*] and [*i*, *j* + 1, *k*], to join the neighbours to one group. Typical six-neighbours erosion is applied thereafter. These minimal erosion operations ensure that direct neighbours of threshold-masked voxels, potentially containing disturbing field gradients, are excluded.

Finally, DIPF estimates a pseudo-susceptibility of the excluded areas and thus the field distortions generated thence. Ideally, the remnant field will be generated by the susceptibility distribution inside the corrected mask only. The benefit of MUBAFIRE Local is illustrated in the lower row of Fig 2.

### Simulation

A realistic simulation must include long range harmonic variations of the static magnetic field, such as those that can be partly corrected by shimming, as well as field shifts induced by susceptibility interfaces at the surface of the observed object and by local (internal) susceptibility contrast. We present a Monte Carlo simulation in which we cover a range of external and internal sources of field distortions to assess performance and accuracy of the presented method.

The field distortion induced by an arbitrary susceptibility distribution, *b*_sus_, can be approximated by a dipole convolution as in Eq 2 [30, 31]. The underlying static field, *B*_0_, is split into a constant component and a spatially varying part, *B*_0_ → *B*_0_(**r**) = *B*_base_ + *b*_inh_(**r**), including long-range inhomogeneities. Inserting this into Eq 2 results in the expression:

$$\begin{array}{ccc} b_{\text{sus}}\left(r\right) & ≔ & B_{\text{true}}\left(r\right)-B_{\text{base}}\\ & = & [B_{\text{base}}+b_{\text{inh}}\left(r\right)]\cdot \left(d*\chi \right)\left(r\right)+b_{\text{inh}}\left(r\right), \end{array}$$ (6)

where the left hand side stands for the simulated field shift (corresponding to the observable shift).

Varying long-range inhomogeneities, *b*_inh_, are simulated by generating SSH functions (see Appendix) with randomised coefficients and polarity. The magnitude of these shifts is restricted to ±400 Hz. The $T_{2}^{*}$ distribution of a *post mortem* brain, measured with a gradient-echo multiple echo sequence, serves as a template for a susceptibility distribution *χ*_stat_, using values in a range of −9±0.2 ppm. Brain segmentation is performed on the same data, generating a brain mask, *m*, and roughly separating brain tissue from blood vessels and from parts of the ventricular system. For the blood vessels, *χ*_stat_ is set to −7.9 ppm whilst the average value of −9 ppm is assigned to the ventricles. The brain exterior is set to *χ*_ext_ = −6 ppm, generating long-range field distortions due to the anatomical form of the brain. The observed internal field is diversified by adding a random susceptibility map, *χ*_var_ to *χ*_stat_, featuring continuous areas and susceptibility surfaces. The randomisation range is ±0.2 ppm and ensures that the internal contrast varies throughout the samples. Furthermore, some areas of positive susceptibility are added to *χ*_var_ on the outside of the mask, representing air cavities with distorting effect on the observed internal field.

The simulated susceptibility distribution, *χ* = *χ*_stat_ + *χ*_var_, and the distorted field, *b*_inh_, are inserted into Eq 6. Selecting a base field strength of *B*_base_ = 9.4T and adding Gaussian noise, *b*_*σ*_(**r**) with *σ* = 0.3 Hz yields the observed field:

bobs(r)=[Bbase+binh(r)]·(d*[χstat(r)+χvar(r)]∑)︸bint(r)+binh(r)+bσ(r). (7)

An image matrix of 138 × 162 × 106 is simulated and the brain size is kept well below the volume size to reduce aliasing and numerical edge artefacts in the DIPF correction.

For SPHINX, DIPF and MUBAFIRE a parameter analysis is performed using the described simulation scheme with 10 different configurations of *b*_inh_ and *χ*_var_.

**Fig 4:**
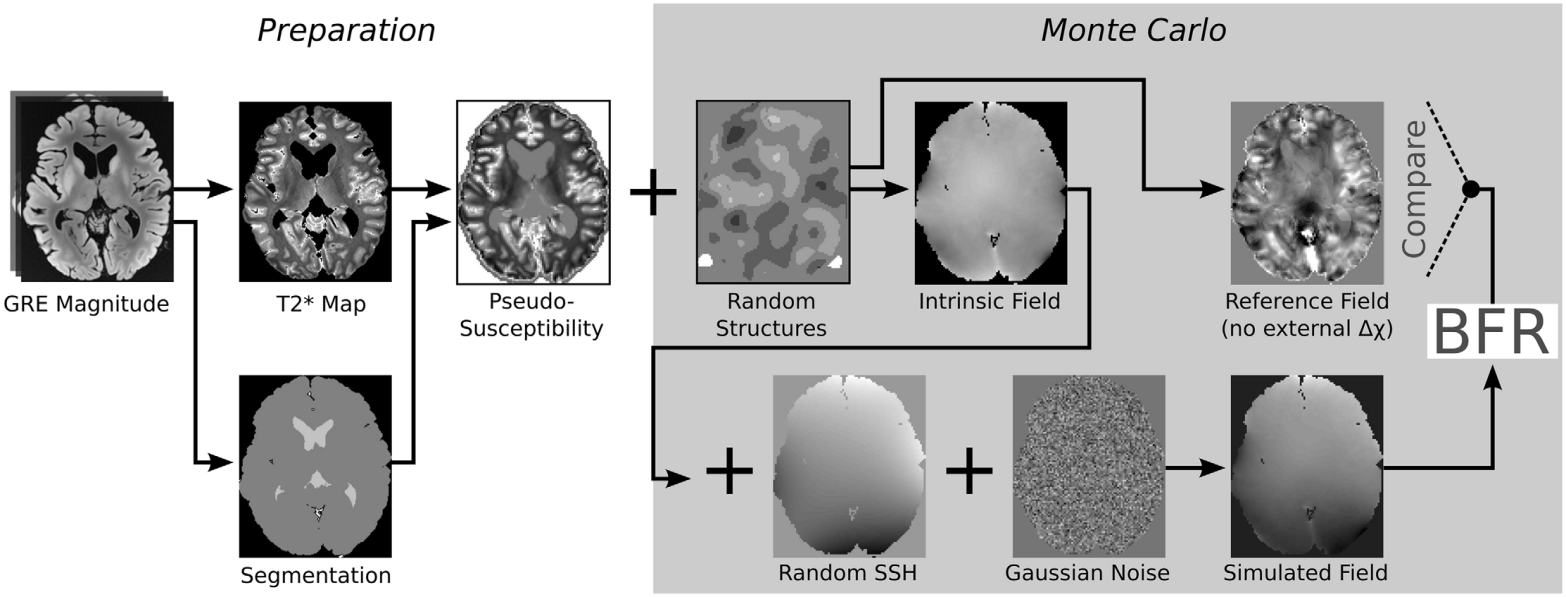
Simulation scheme. A gradient echo measurement is used to estimate a $T_{2}^{*}$ map. Using the $T_{2}^{*}$ anatomy and rescaling the values to an appropriate range, a susceptibility distribution is generated. Random susceptibility structures are added and dipole convolution generates the susceptibility-induced field map and a local reference. External disturbances are introduced as randomised spherical harmonic functions and noise is added. After BFR correction the results are compared to the reference field.

The simulated field is finally analysed by all BFR algorithms introduced above. A set of 50 samples ensures that the outcome is statistically representative. The simulation procedure is described schematically in Fig 4. As a reference, an internal field map, *b*_int_(**r**), is calculated by evaluating only the convolution term in Eq 7 while setting $\chi_{\text{ext}}=\bar{\chi (r)}$ (the average internal susceptibility value) in the exterior. This map represents the (internal) local field contrast.

### MR Measurements

Measurements were performed a) on a 3T Tim Trio scanner and b) and c) on a 9.4T human MR scanner (both: Siemens Healthcare, Erlangen, Germany). In case a), a twelve-channel phased array coil was used to scan ten healthy volunteers. A standard 3D gradient echo sequence with slab-selective excitation was employed. Sequence parameters included: *TR* = 51 ms, *TE* = [2.58, 6.57, 10.53, 14.52, 18.48, 22.47, 26.43, 30.42, 34.38, 38.37, 42.33, 46.32] ms, *α* = 8°, 1 average, *BW* = 260 Hz/Px, 1 mm isotropic resolution with an imaging matrix of 156 × 192 × 128 (transverse slicing). The entire brain was covered by the FOV. A brain mask was generated with *bet2* [38]. In case b), an eight-channel coil was used in single-channel transmit and eight-channel receive mode to measure the fixed brain. In order to maximise the accuracy and spatial consistency of the phase data only the channel with highest signal-to-noise ratio (SNR) was used for further analysis. A slab-selective 3D gradient echo sequence was employed. Sequence parameters included: *TR* = 63 ms, *TE* = 16.38 ms, *α* = 25° (nominal), 12 averages, *BW* = 40 Hz/Px and an image matrix of 480 × 512 × 160 (sagittal slicing) at 240 *μ*m isotropic resolution. Parts of the central brain, the brain stem and the cerebellum were covered by the measurement. A VOI (175 × 285 × 111 voxels) with high receive sensitivity was chosen for further analysis.

The ultra-high field *in vivo* measurement c) includes a standard gradient echo, multiple echo sequence using *TR* = 38 ms, *TE* = [3.93, 9.68, 15.43, 21.18, 26.93] *ms* (only the first three echoes were used), *α* = 48° (nominal), 1 average, *BW* = 235 Hz/Px and matrix size 308 × 448 × 80 at 0.5 mm isotropic resolution. The data of two different transmit configurations were joined by combining the receive channels, normalised to the first echo, in each measurement and applying magnitude-weighted averaging of the field maps acquired from both measurements.

At the beginning of each measurement, 3D shimming was performed with the manufacturer-supplied procedure used iteratively (4–8 times) until the full width half maximum (FWHM) of the signal from the whole head converged. The shim values were applied to all subsequent scans.

### Parameters and Evaluation

Phase data are unwrapped using either PRELUDE [25] or an in-house method called URSULA [39], respectively. In case a) and c) field maps are calculated by linear regression of the unwrapped phase, whilst the single-echo case b) requires only division by the echo time.

The experimental data are corrected with GF, SPHINX, DIPF, MUBAFIRE and MUBAFIRE Local. All presented algorithms were implemented in Matlab (The Mathworks Inc., Natick, USA). The parametrisation for GF is heuristically chosen, but defined in comparable dimensioning with respect to the brain size for the simulation and the *in vivo* study: *σ* = 4 is used for the synthetic data, while in a) *σ* = 6 and in b) and c) *σ* = 8. DIPF is applied with zero padding of 1/8 of the full resolution, *λ* = 500 and 50 iterations. This includes its application in MUBAFIRE and MUBAFIRE Local. The SPHINX correction is applied at an order of 10 standalone, and at an order of 4 within MUBAFIRE. Thresholding within MUBAFIRE Local is performed with *n*_*σ*_ = 8 for the study a) and measurement b), while *n*_*σ*_ = 14 is used for c).

In the parameter optimisation, standalone SPHINX is applied with 1^*st*^ to 15^*th*^ order, DIPF is applied with 5, 10, 20, …, 200 iterations. In the context of MUBAFIRE, SPHINX is applied with 1^*st*^ to 10^*th*^ order, DIPF is parametrised as before. The simulation does not produce voxels exceeding measurement range or giving erroneous information. Consequently, MUBAFIRE Local is only applied on measured data, but not on the synthetic data. The simulation BFR results are evaluated by calculating the L_1_-norm of the difference between the BFR results and the mean-corrected reference (true) field, *b*_int_, inside the brain mask:

$$\begin{array}{ccc} L_{1}(\Delta b) & = & \frac{1}{n_{\text{voxels}}}\cdot \sum_{\forall r\in m}\;[b_{\text{corr}}\left(r\right)-b_{\text{ref}}\left(r\right)],\\ \text{where}:\;b_{\text{ref}} & = & b_{\text{int}}-\bar{b_{\text{int}}}, \end{array}$$ (8)

and $\bar{b_{\text{int}}}$ is the average of *b*_int_ inside the brain mask. Furthermore, the histograms and the standard deviation of the field distributions inside the brain mask are compared and visual inspection of the image contrast is performed.

All measurements are evaluated by comparing the standard deviation and visual contrast of the corrected maps. This approach is suggested by the results of the simulation (see below).

## Results

**Fig 5:**
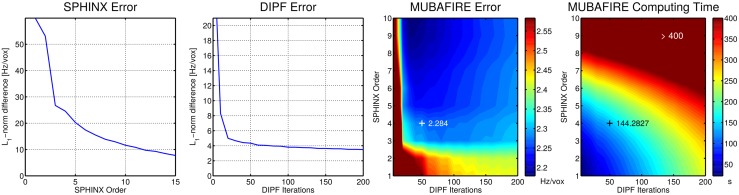
Parameter Optimisation. From left to right, the resulting error for standalone SPHINX, DIPF and MUBAFIRE are shown for a range of parameterisations. The rightmost graph illustrates the required computing time for MUBAFIRE.

The parameter optimisation is illustrated in Fig 5, showing the normalised result error for SPHINX with increasing harmonic order, as well as for DIPF with increasing iteration count. With SPHINX, the error decreases continuously, even at orders higher than ten. The curve of DIPF starts with a steep descent and the improvements for orders higher than 25 become more and more marginal. The normalised error and computing time shown on the right hand side of Fig 5 describe the behaviour within MUBAFIRE for different parameter sets, always including an initial linear correction. Choosing order 4 in SPHINX and 50 iterations in DIPF offers a good compromise between result error and computing time demands.

**Fig 6:**
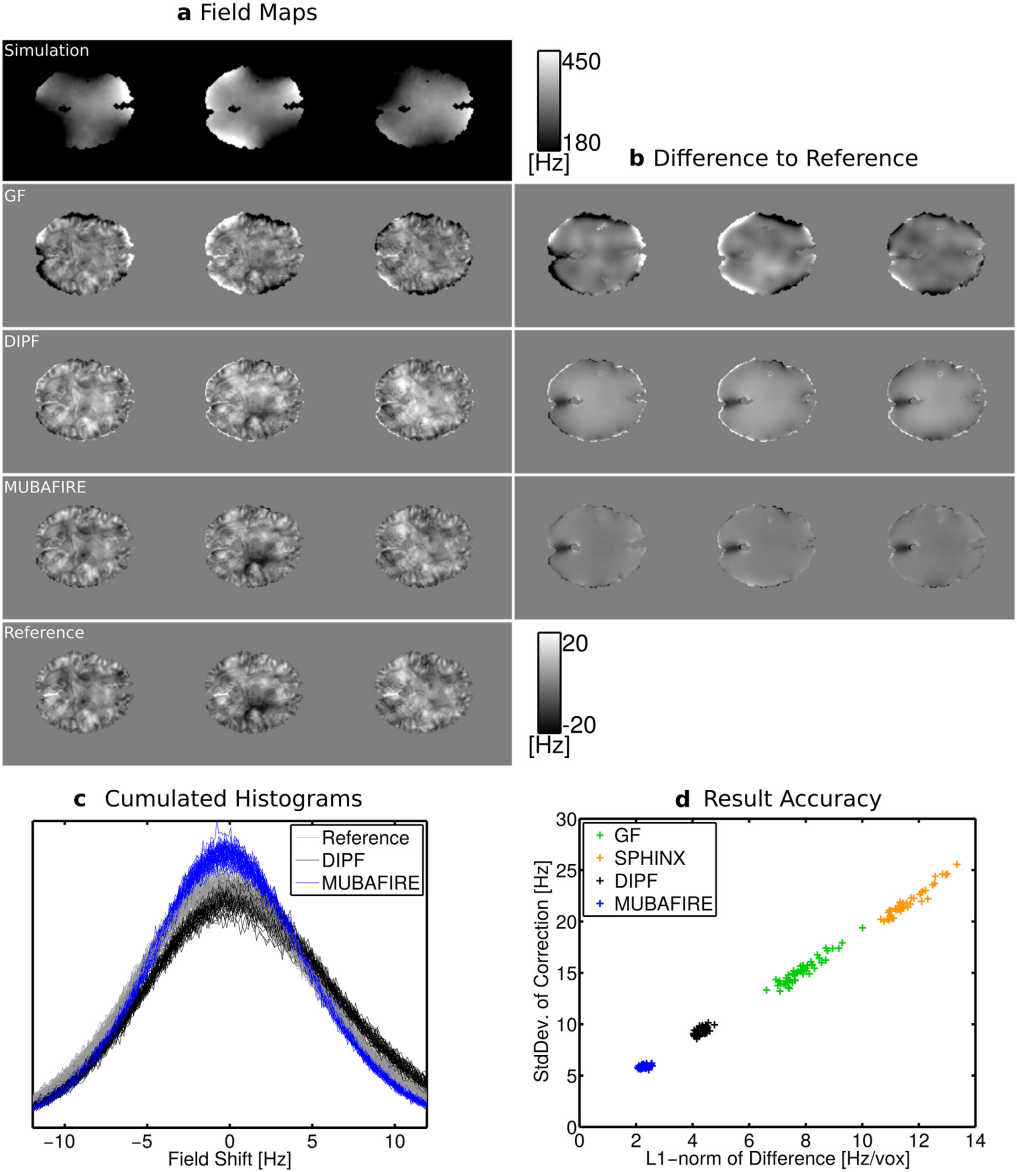
Results of the BFR applied to the numerical data. (a) showing from left to right three simulation samples and from top to bottom: raw simulation, GF, DIPF, MUBAFIRE correction and the reference field, *b*_ref_; (b) shows the difference between corrected fields and *b*_ref_. The cumulated DIPF and MUBAFIRE histograms of all 50 samples are shown in (c) in comparison to the reference. Finally, (d) shows correlation between L_1_-norm and standard deviation of GF, SPHINX, DIPF and MUBAFIRE correction.

In Table 1 the results of all BFR algorithms applied to the Monte Carlo simulation are illustrated. The table includes the 𝕃_1_-norm of the difference between corrected and reference field averaged over all simulation samples. Further, the standard deviation of this value is reported. Whilst GF shows a large deviation from the reference, DIPF performs distinctly better. Standalone SPHINX generates high standard deviation and error level. MUBAFIRE substantially outperforms DIPF in terms of accuracy, reducing the difference norm by almost 50%. For DIPF and MUBAFIRE, the standard deviation of the statistics remains below 7% of the average value. Further, the average of all standard deviation values of the corrected field maps throughout the simulation samples is shown, as well as its variation (standard deviation). The GF exhibits a more than two times higher average standard deviation than the reference, while DIPF only deviates by about 50%. The closest match is achieved by MUBAFIRE with 6% underestimation. The values vary little throughout the samples, leading to *σ*_*n*_ ≤ 10% for GF, *σ*_*n*_ < 4% for DIPF and for MUBAFIRE even *σ*_*n*_ ≈ 3%. Besides sample slices, Fig 6 shows the cumulated view of the histograms from all cases corrected with MUBAFIRE and DIPF in relation to the reference field histogram. Although the histograms differ only slightly, MUBAFIRE shows a higher similarity to the reference than DIPF, the latter exhibiting slight shifts towards the positive frequency range. A plot of the standard deviation and the 𝕃_1_-norm of GF, SPHINX, DIPF and MUBAFIRE results shows a correlation between both measures.

**Table 1:** Results of BFR on Monte Carlo Simulation.

**Correction Results for Monte Carlo Simulation (*n* = 50)**					
	**GF**	**SPHINX**	**DIPF**	**MUBAFIRE**	**Reference**
mean_*n*_[𝕃_1_(Δ*b*)]	7.86	11.55	4.32	2.29	(0.0)
±*σ*_*n*_[𝕃_1_(Δ*b*)]	0.67	0.66	0.16	0.14	(0.0)
mean_*n*_[*σ*(*b*)]	15.19	21.71	9.34	5.86	6.25
±*σ*_*n*_[*σ*(*b*)]	1.30	1.37	0.33	0.14	0.14

Units are Hz. The rows show mean and standard deviation of the 𝕃_1_-norm determined over *n* = 50 simulations. Further the mean (over *n*) standard deviation and its standard deviation are evaluated.

**Fig 7:**
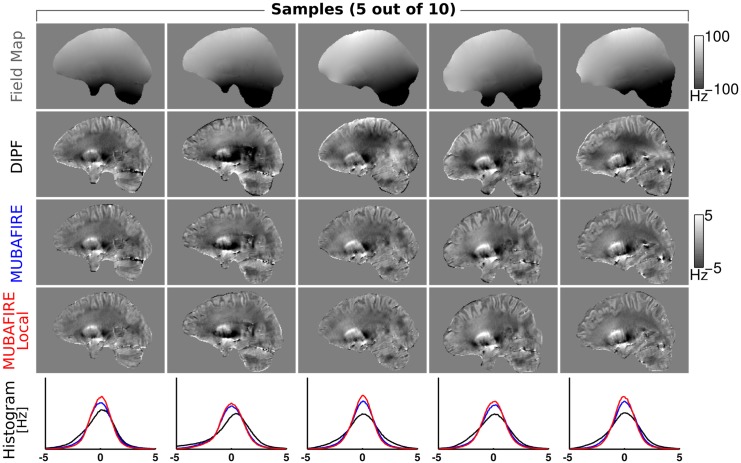
Measurement series at 3T. From top to bottom the field map, DIPF, MUBAFIRE and MUBAFIRE Local correction for five samples of the 3T study are shown. The bottom row illustrates histograms of the field distribution for the correction algorithms in each sample.

**Fig 8:**
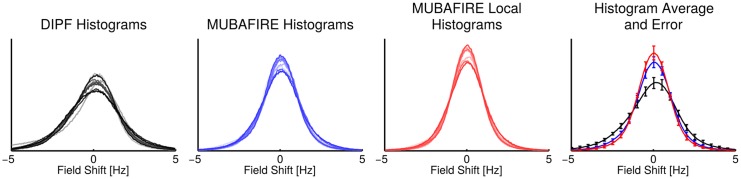
Cumulated histograms of the measurement series. From left to right: Cumulated DIPF, MUBAFIRE and MUBAFIRE Local histograms of 3T *in vivo* study—different shadings indicate the cases. The rightmost graph shows the average distribution of all cases, error bars indicate the standard deviation.

Fig 7 illustrates non-coregistered, central sagittal views of the field map for five representative cases of the 3T measurement series. The DIPF, MUBAFIRE and MUBAFIRE Local corrections are shown as well as histograms with the corresponding field distribution. In all cases MUBAFIRE shows a higher and narrower distribution than DIPF; MUBAFIRE Local indicates even more improvements. Local contrast—as determined visually—is preserved between these algorithms, yet DIPF shows slight over- and underestimations in the mask centre. In Fig 8 the histograms for all cases are plotted in cumulated view and a mean histogram illustrates the average and standard deviation of the field distributions for the algorithms. The average confirms the observations of the individual cases. Table 2 contains the numerical results of all cases and indicates the same decrease in the standard deviation from DIPF to MUBAFIRE and finally to MUBAFIRE Local. In contrast to the simulation, the GF shows a lower standard deviation than DIPF for several cases and in the average.

**Table 2:** Results of BFR on 3T measurement series.

**Correction results measurement series 3 T**					
	**GF**	**SPHINX**	**DIPF**	**MUBAFIRE**	**M. Local**
*σ*(*b*_*n*_)	2.96	3.77	2.37	1.63	1.22
cases	2.92	3.78	6.43	1.75	1.48
*n* = 1–10	3.04	4.32	2.90	1.53	1.25
…	2.70	3.66	3.18	1.60	1.33
	2.87	4.18	4.45	1.50	1.22
	2.94	4.74	4.22	1.62	1.25
	3.11	3.85	1.95	1.57	1.28
	3.19	4.12	2.50	1.76	1.43
	2.86	3.88	5.60	1.75	1.26
	2.94	3.65	3.62	1.85	1.51
mean_*n*_(*σ*(*b*_*n*_))	2.95	4.00	3.72	1.66	1.32
±*σ*_*n*_(*σ*(*b*_*n*_))	0.14	0.35	1.46	0.12	0.11

**Fig 9:**
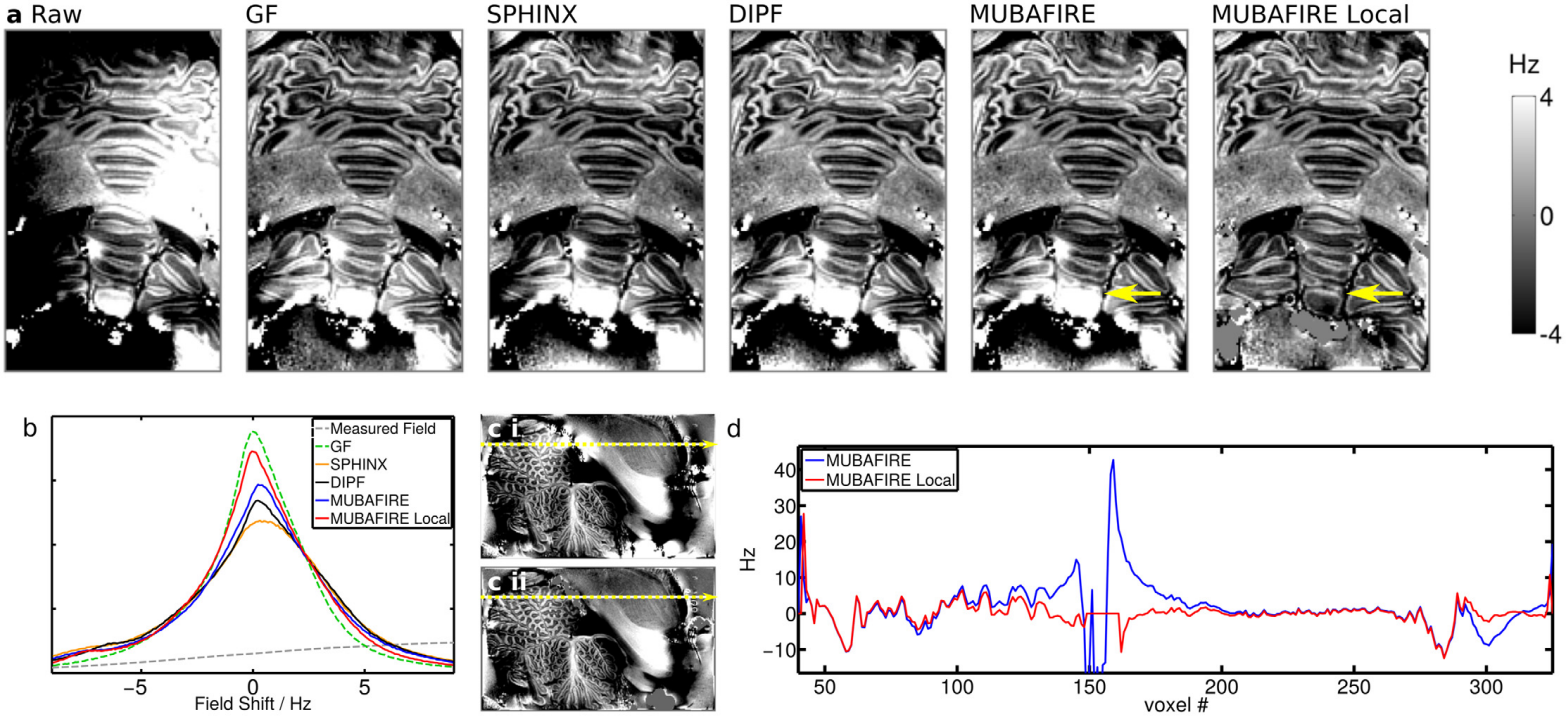
Results of the BFR algorithms applied on measurement b) (*post mortem* at 9.4T). (a) shows a frontal sample slice of the VOI, from left to right: raw, GF, SPHINX, DIPF, MUBAFIRE, MUBAFIRE Local; yellow arrows indicate differences; (b) histogram comparison; (c i-ii) position of the line plot in MUBAFIRE (c i) and MUBAFIRE Local (c ii) corrected slice; (d) line plot.

**Fig 10:**
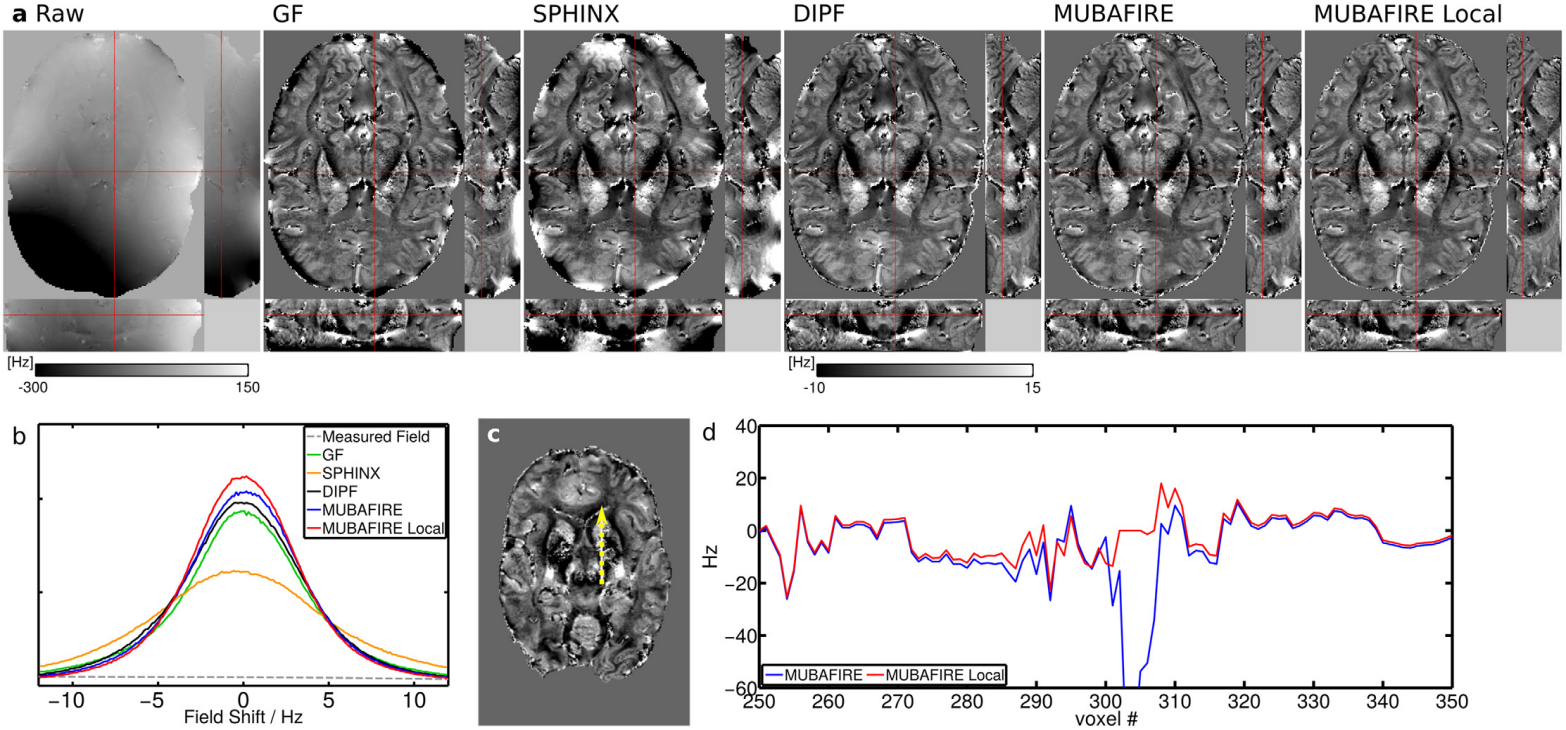
Measurement c): *in vivo* BFR results at 9.4T. (a) shows orthogonal sample slices, from left to right: raw, GF, SPHINX, DIPF, MUBAFIRE, MUBAFIRE Local; (b) histogram overview; (c) shows position of the line plot in the MUBAFIRE Local correction and (d) the according line plot comparing MUBAFIRE and MUBAFIRE Local in magnified view.

**Fig 11:**
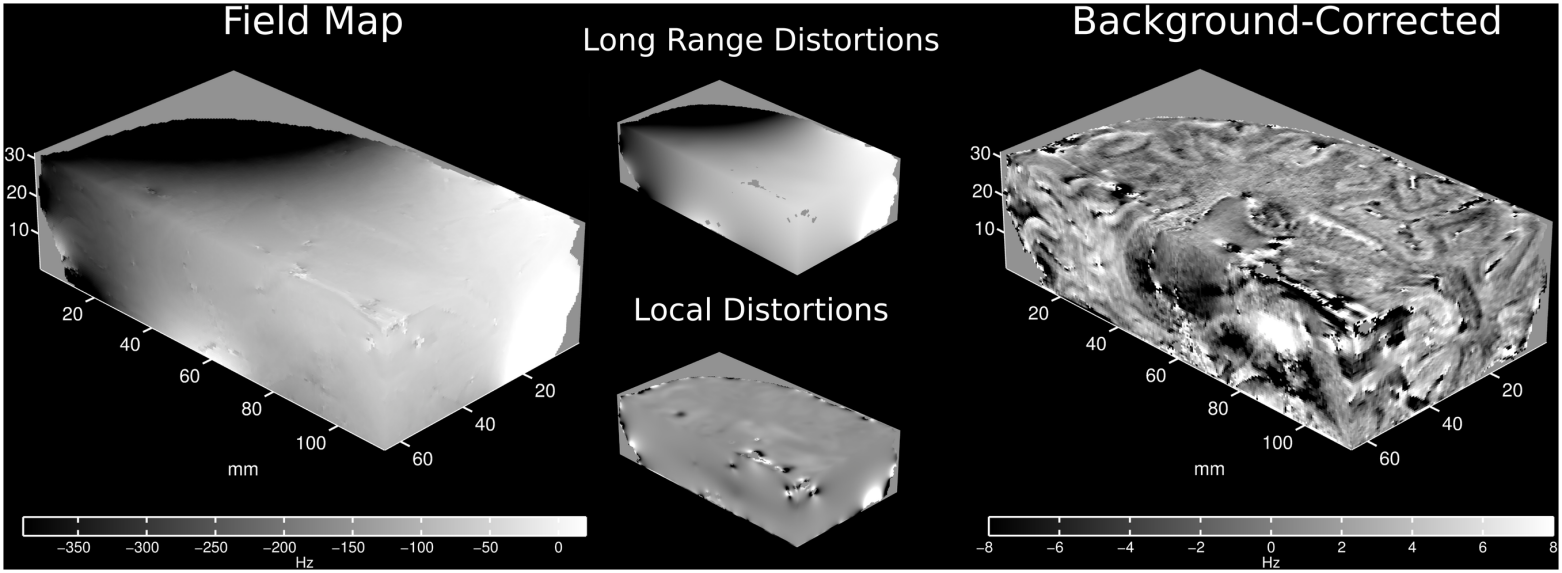
Spatial view of BFR on 9.4T *in vivo* measurement. Actual field map (left) and MUBAFIRE Local corrected field (right) recorded and computed from the 9.4T *in vivo* measurement. In the middle, long-range distortions (MUBAFIRE) and local distortions (MUBAFIRE Local) are visualised.

For the 9.4T measurements b) and c), representative slices were chosen and plotted for each individual BFR algorithm, separately. This is shown in Figs 9 and 10. A magnified view of the raw field map and the MUBAFIRE Local correction for the *in vivo* case is illustrated in Fig 11. A histogram is plotted for each experiment, describing the field distribution inside the brain mask. For the *post mortem* brain measurement, GF shows the highest peak followed by MUBAFIRE Local. For the *in vivo* measurement, MUBAFIRE Local shows the narrowest field distribution. Mean and standard deviation are lower for MUBAFIRE in comparison to the DIPF (see Table 3). This observation is in good agreement with the simulation results.

**Table 3:** Results of BFR on 9.4T measurements.

**Correction results for cerebellum at 9.4T**					
	**GF**	**SPHINX**	**DIPF**	**MUBAFIRE**	**M. Local**
mean(*b*)	0.0185	-0.0154	0.0963	0.0337	0.0737
*σ*(*b*)	5.7816	6.8894	6.4352	6.3977	4.6326
**Correction results for 9.4T** ***in vivo*** **measurement**					
	**GF**	**SPHINX**	**DIPF**	**MUBAFIRE**	**M. Local**
mean(*b*)	0.0537	-0.0133	-0.2305	0.0185	0.1112
*σ*(*b*)	13.2919	16.9396	9.4057	8.9163	8.8527

Units are Hz. The mean field and standard deviation of the corrected field from the measurements are shown.

The benefit of the MUBAFIRE Local filter at 9.4T is outlined in line plots (Figs 9 and 10). The line plot in Fig 9 shows a strong spike. Judging from the image, the responsible structure appears to be a dipole-like distortion, most likely due to the presence of an air bubble adjacent to fixed brain tissue.

## Discussion

The simulation results in Table 1 and Fig 6 demonstrate that the GF generates reduced contrast and strong edge artefacts. These artefacts are also responsible for the high standard deviation values observed for the GF that accompany the low visual contrast. Harmonic filtering with SPHINX only moderately decreases the field inhomogeneity when applied standalone, reflecting the fact that only long-range distortions can be filtered herewith.

More importantly, the simulation shows that MUBAFIRE performs more accurately than DIPF. This is indicated by the significantly lower 𝕃_1_-norm, from the fact that the value of the standard deviation approaches that of the reference field and from the features of the field distribution as reflected by the histograms. Compared to the histogram of the true field distribution, DIPF generates significant shifts and deviations whereas MUBAFIRE lies closer to the true distribution with a slight overestimation around zero. Although the visual image contrast (in the sample slices) seems to be preserved, inaccuracies of the DIPF appear as slight under- and overestimations at mask rim and centre. These correspond to the observed histogram displacement. The deviations appear distinctly improved in the MUBAFIRE slices.

The correlation plot indicates that relating the standard deviation of the correction result to the accuracy seems a promising approach—a decreased standard deviation appears to suggest a higher fit accuracy for the algorithms under investigation. Since the decrease can also be caused by loss in contrast, as for central regions of the GF result, this measure must be treated with care, and should include visual inspection of the corrected data.

The error in the parameter optimisation, illustrated in Fig 5, is relatively high. Yet, one has to consider that the 𝕃_1_-norm is calculated from the raw results, including potential constant offsets between ground truth and correction. The results imply that MUBAFIRE is not only more precise than applying common DIPF, even up to a high order, but also more time efficient. The accuracy of standalone SPHINX can be significantly increased by using higher harmonic orders but, when combined within MUBAFIRE, lower orders and iteration counts suffice for high result accuracy and demand only moderate computing time. For high spherical harmonic orders, lower DIPF iteration counts lead to the highest result precision. This is most likely due to over-compensation effects of the DIPF.

The numerical results of the *in vivo* study reflect the simulation results regarding the behaviour of the standard deviation of DIPF and MUBAFIRE. The visual results indicate that, as observed in the simulation, standalone DIPF tends to generate slight under- or overestimation in central FOV regions, while MUBAFIRE produces a more homogeneous outcome under preservation of the image contrast. Besides demonstrating the benefit of MUBAFIRE, in particular the visual results show the advantages of additionally running MUBAFIRE Local in the removal of local perturbation that exceed the normal contrast range and the correction of their influences on surrounding regions. Interestingly, several cases and even the average over all cases show a standard deviation for GF that is lower than for the DIPF. Hence, either GF leads to better or DIPF to less accurate results than in the simulation. The reason for this is not evident, but might originate in the masking process. However, this has no impact on the positive results for MUBAFIRE.

The sample slices for the measurements b) and c) illustrate the characteristics of the algorithms under differing conditions. As expected, GF produces significant artefacts at the mask edges. The low standard deviation, compared to the other filters, originates in the poor contrast and the lower volume fraction occupied by the edge artefacts in comparison to the simulation. The filter has little physical basis and cannot distinguish between long to medium size field distortions induced by outside sources and contrast originating from inside the VOI on a medium-size scale. The standalone SPHINX results in significantly higher image contrast compared to GF, even though no local distortions are considered. DIPF and MUBAFIRE (Local) visually show the greatest richness in overall contrast. The standard deviation of the corrected field confirms these observations—The lower standard deviation of MUBAFIRE and the preserved visual contrast indicate that the filter performs more accurately than DIPF.

The standard deviation for MUBAFIRE Local is in the same range as for MUBAFIRE in measurement c), but not in b), where it becomes significantly smaller. The most probable reason for this lies in the existence of small air bubbles in *post mortem* tissue which create a strong field disturbance and are successfully removed by MUBAFIRE Local. Their effect is also expected to be stronger at 9.4T than at 3T. The line plot of case b) (Fig 9) illustrates that MUBAFIRE Local triggers significant improvement of these local distortions, while keeping the structural profile of the field unchanged. Also, in measurement c), it is possible to compensate for local, potentially noise-related distortions (Fig 10).

The local correction involves a removal of voxels by thresholding and erosion and can occasionally render the representation of small anatomical structures incomplete. Nevertheless, the excluded voxels do not contain representative data, e.g. because their field values are falsified by intra-voxel gradients. This makes the MUBAFIRE Local step greatly profitable.

Since the DIPF correction is generally considered the gold standard for BFR and is based on sound physical principles, the superior performance of MUBAFIRE versus DIPF deserves further attention. Theoretically, the DIPF filter is able to remove long-range harmonic distortions. However, the superposition of dipole fields is not the appropriate description of a field generated by sources which are not included in the reconstruction FOV. In practice, the method suffers from insufficient volume size, a finite iteration count and it lacks a minimisation strategy optimised for distant sources of field shifts. In our experience, the performance of DIPF improves with increasing iteration count and for extensive zero padding (several multiples of the object size to be analysed). The associated computation demands are high and can be avoided by a problem-adapted technique such as MUBAFIRE. In other words, the SPHINX correction in MUBAFIRE simply provides an appropriate basis to describe fields produced by sources outside the FOV.

Of course, this technique still has limitations—high harmonic orders cannot be included in a time-efficient implementation. Hence, besides numerical errors in the DIPF, external sources that are expressed in high order harmonic characteristics cannot be removed completely. Nevertheless, the standalone filters suffer from similar problems. Thus, MUBAFIRE appears superior regarding the better correction results attained.

## Conclusions

In conclusion, we have introduced a novel BFR algorithm, MUBAFIRE, which offers a robust means to correct field maps for arbitrary background shifts. Its performance was evaluated based on numerical simulations. Also, the parameterisation of the algorithm was optimised in the context of this simulation. Using the visible contrast and the field standard deviation as indicators, it was found that MUBAFIRE performs more accurately than DIPF in the presented scenarios. The observations were successfully validated in the context of an *in vivo* study on ten volunteers. Analysis of 9.4T measurement data supports these results. The standard deviation of the calculated field distribution is smaller and the contrast is preserved when MUBAFIRE is used. Externally and internally induced shifts can be addressed as well as static field variations (see Fig 11). Especially at ultra-high field strengths, such as 9.4T, field shifts induced by small vessels, air cavities or other material may exceed the field range that can be accurately measured given the experimental parameters or generate intra-voxel field gradients. MUBAFIRE Local provides an adequate way to remove such erroneous field values from the data and to compensate for their influences on the entire map.

While relying solely on physically meaningful models for background field correction, MUBAFIRE (Local) produces field maps of great local detail—such as the clearly delineated cerebellar cortex in measurement b) or the contrast inside the basal ganglia in case c). The algorithm is geared to the needs of studies investigating phase contrast and field information, but the final aim is to apply it to quantitative magnetic susceptibility mapping. The latter application strongly benefits from the use of physically correct maps of the local magnetic field produced by tissue such as those that MUBAFIRE can provide. Investigation of the algorithm in a susceptibility reconstruction framework is our next objective.

## Appendix

The real regular solid spherical harmonics can be expressed as $v_{lm}\left(\vec{r}\right)=N_{lm}\cdot r^{l}\cdot Y_{lm}\left(\theta ,\phi \right)$, where **r** = (*r*, *θ*, *ϕ*) are the spherical coordinates, *l* represents the order and *m* ∈ ℕ is confined to: *m* ∈ [−*l*, …, −1, 0, 1, …, *l*]. Further, *Y*_*lm*_(*θ*, *ϕ*) are the simple spherical harmonic functions in spherical notation (see [36]).

The orthonormalised SSHs are defined as follows:

$${\hat{u}}_{00}=\frac{v_{00}}{|v_{00}|},$$ (9)

$$\begin{array}{cc} {\hat{u}}_{lm}=v_{lm} & -\sum_{j=0}^{l-1}\sum_{k=-j}^{j}\left\langle {\hat{u}}_{jk}|v_{lm}\right\rangle \cdot {\hat{u}}_{jk}\\ & -\sum_{k=-l}^{m}\left\langle {\hat{u}}_{lk}|v_{lm}\right\rangle \cdot {\hat{u}}_{lk}. \end{array}$$ (10)

The modified SSHs, ${\hat{u}}_{lm}$, fulfill:

$$\left\langle {\hat{u}}_{lm}|{\hat{u}}_{no}\right\rangle =\delta_{ln}\delta_{mo},$$ (11)

where 〈∣〉 is the internal (voxelwise) product and *δ*_*ij*_ represents the Kronecker delta symbol.

## References

[1] Lai S, Reichenbach JR, Haacke EM. Commutator filter: a novel technique for the identification of structures producing significant susceptibility inhomogeneities and its application to functional MRI. Magnet Reson Med. 1996 11;36(5):781–7. Available from: http://www.ncbi.nlm.nih.gov/pubmed/8916030. 10.1002/mrm.1910360518 8916030

[2] Reichenbach JR, Venkatesan R, Yablonskiy DA, Thompson SL, Haacke EM. Theory and application of static field inhomogeneity effects in gradient-echo imaging. JMRI-J Magn Reson Im. 1997;7(2):266–279. 10.1002/jmri.1880070203 9090577

[3] Haacke EM, Xu Y, Cheng YCN, Reichenbach JR. Susceptibility weighted imaging (SWI). Magnet Reson Med. 2004 9;52(3):612–8. Available from: http://www.ncbi.nlm.nih.gov/pubmed/15334582. 10.1002/mrm.20198 15334582

[4] de Rochefort L, Liu T, Kressler B, Liu J, Spincemaille P, Lebon V, et al Quantitative susceptibility map reconstruction from MR phase data using bayesian regularization: validation and application to brain imaging. Magnet Reson Med. 2010 1;63(1):194–206. Available from: http://www.ncbi.nlm.nih.gov/pubmed/19953507. 10.1002/mrm.22187 19953507

[5] Shmueli K, de Zwart Ja, van Gelderen P, Li TQ, Dodd SJ, Duyn JH. Magnetic susceptibility mapping of brain tissue in vivo using MRI phase data. Magnet Reson Med. 2009 12;62(6):1510–22. Available from: http://www.ncbi.nlm.nih.gov/pubmed/19859937. 10.1002/mrm.22135 PMC4275127 19859937

[6] Wharton S, Bowtell R. Whole-brain susceptibility mapping at high field: a comparison of multiple- and single-orientation methods. NeuroImage. 2010 11;53(2):515–25. 10.1016/j.neuroimage.2010.06.070 20615474

[7] Wharton S, Schäfer A, Bowtell R. Susceptibility mapping in the human brain using threshold-based k-space division. Magnet Reson Med. 2010 5;63(5):1292–304. Available from: http://www.ncbi.nlm.nih.gov/pubmed/20432300. 10.1002/mrm.22334 20432300

[8] Liu C. Susceptibility tensor imaging. Magnet Reson Med. 2010 6;63(6):1471–7. Available from: http://www.pubmedcentral.nih.gov/articlerender.fcgi?artid=2990786&tool=pmcentrez&rendertype=abstract. 10.1002/mrm.22482 PMC2990786 20512849

[9] Liu J, Drangova M. Phase-unwrapping algorithm for translation extraction from spherical navigator echoes. Magnet Reson Med. 2010 2;63(2):510–6. Available from: http://www.ncbi.nlm.nih.gov/pubmed/19918896. 10.1002/mrm.22198 19918896

[10] Langkammer C, Krebs N, Goessler W, Scheurer E, Yen K, Fazekas F, et al Susceptibility induced gray-white matter MRI contrast in the human brain. NeuroImage. 2012 1;59(2):1413–9. Available from: http://www.pubmedcentral.nih.gov/articlerender.fcgi?artid=3236994&tool=pmcentrez&rendertype=abstract. 10.1016/j.neuroimage.2011.08.045 21893208 PMC3236994

[11] Duyn JH, van Gelderen P, Li TQ, de Zwart Ja, Koretsky AP, Fukunaga M. High-field MRI of brain cortical substructure based on signal phase. P Natl Acad Sci USA. 2007 7;104(28):11796–801. Available from: http://www.pubmedcentral.nih.gov/articlerender.fcgi?artid=1913877&tool=pmcentrez&rendertype=abstract. 10.1073/pnas.0610821104 PMC1913877 17586684

[12] Schenck J. The role of magnetic susceptibility in magnetic resonance imaging: MRI magnetic compatibility of the first and second kinds. Med Phys. 1996;23:815–850. Available from: http://ee-classes.usc.edu/ee591/library/Schenck-Suscept.pdf. 10.1118/1.597854 8798169

[13] Li S, Dardzinski BJ, Collins CM, Yang QX, Smith MB. Three-dimensional mapping of the static magnetic field inside the human head. Magnet Reson Med. 1996 11;36(5):705–14. Available from: http://www.ncbi.nlm.nih.gov/pubmed/8916021. 10.1002/mrm.1910360509 8916021

[14] Koch KM, Papademetris X, Rothman DL, de Graaf Ra. Rapid calculations of susceptibility-induced magnetostatic field perturbations for in vivo magnetic resonance. Phys Med Biol. 2006 12;51(24):6381–402. 10.1088/0031-9155/51/24/007 17148824

[15] Marques JPRF. Effects of Dipolar Fields in NMR and MRI [PhD Thesis]. University of Nottingham; 2004.

[16] Abduljalil AM, Schmalbrock P, Novak V, Chakeres DW. Enhanced gray and white matter contrast of phase susceptibility-weighted images in ultra-high-field magnetic resonance imaging. JMRI-J Magn Reson Im. 2003 9;18(3):284–90. Available from: http://www.ncbi.nlm.nih.gov/pubmed/12938122. 10.1002/jmri.10362 12938122

[17] de Rochefort L, Delzor A, Guillermier M, Houitte D, Chaigneau M, Déglon N, et al Quantitative Susceptibility Mapping In Vivo in the Rat Brain. Proc Intl Soc Magnet Reson Med. 2009;17(c):1134.

[18] Liu T, Khalidov I, de Rochefort L, Spincemaille P, Liu J, Tsiouris AJ, et al A novel background field removal method for MRI using projection onto dipole fields (PDF). NMR Biomed. 2011;24(9):1129–1136. 10.1002/nbm.1670 21387445 PMC3628923

[19] Schweser F, Deistung A, Lehr BW, Reichenbach JR. Quantitative imaging of intrinsic magnetic tissue properties using MRI signal phase: an approach to in vivo brain iron metabolism? NeuroImage. 2011 2;54(4):2789–807. 10.1016/j.neuroimage.2010.10.070 21040794

[20] Sun H, Wilman AH. Background field removal using spherical mean value filtering and Tikhonov regularization. Magnet Reson Med. 2013 5;1157:1151–1157. Available from: http://www.ncbi.nlm.nih.gov/pubmed/23666788. 10.1002/mrm.24765 23666788

[21] Hirsch S, Oros-Peusquens AM, Shah NJ. Phase contrast: describing the global field variations. “High Field Systems & Applications” (Conference Contribution). 2009;p. 31.

[22] Lindemeyer J, Oros-Peusquens AM, Shah NJ. Stepwise filtering of background fields: optimising susceptibility reconstruction. Magnetic Resonance Materials in Physics, Biology and Medicine. 2011;24(1,supplement):507.

[23] Lindemeyer J, Oros-Peusquens AM, Shah NJ. Microstructure Phase Imaging at 9.4T of the Human Brain applying Stepwise Filtering of Background Fields. Proc Intl Soc Magnet Reson Med. 2012;20:2329.

[24] Lindemeyer J. Optimisation of Phase Data Processing for Susceptibility Reconstruction in Magnetic Resonance Imaging. PhD Thesis. RWTH Aachen University. Aachen; 2015. Available from: http://publications.rwth-aachen.de/record/465249.

[25] Jenkinson M. Fast, automated, N-dimensional phase-unwrapping algorithm. Magnet Reson Med. 2003 1;49(1):193–7. Available from: http://www.ncbi.nlm.nih.gov/pubmed/12509838. 10.1002/mrm.10354 12509838

[26] Jezzard P, Balaban R. Correction for geometric distortion in echo planar images from B0 field variations. Magnetic resonance in medicine. 1995;34:65–73. 10.1002/mrm.1910340111 7674900

[27] Bernstein MA, King KF, Zhou XJ. Handbook of MRI Pulse Sequences. Elsevier; 2004.

[28] Reichenbach J, Haacke E. High-resolution BOLD venographic imaging: a window into brain function. NMR Biomed. 2001;p. 453–467. 10.1002/nbm.722 11746938

[29] Hirsch S. Development of a Novel Method for B0 Inhomogeneity Suppression in MRI Field Maps Using Adapted Solid Spherical Harmonic Functions [Diploma Thesis]. Johannes Gutenberg-Universität Mainz; 2009.

[30] Salomir R, de Senneville BD, Moonen CT. A fast calculation method for magnetic field inhomogeneity due to an arbitrary distribution of bulk susceptibility. Concept Magnetic Res. 2003 10;19B(1):26–34. 10.1002/cmr.b.10083

[31] Marques JP, Bowtell R. Application of a Fourier-based method for rapid calculation of field inhomogeneity due to spatial variation of magnetic susceptibility. Concept Magnetic Res Part B: Magnet Reson Eng. 2005 4;25B(1):65–78. 10.1002/cmr.b.20034

[32] Shewchuk JR. An Introduction to the Conjugate Gradient Method Without the Agonizing Pain; 1994. Available from: http://www.cs.cmu.edu/~quake-papers/painless-conjugate-gradient.pdf.

[33] Bouwman JG, Bakker CJG. Alias subtraction more efficient than conventional zero-padding in the Fourier-based calculation of the susceptibility induced perturbation of the magnetic field in MR. Magnet Reson Med. 2012 6;68(2):621–630. 10.1002/mrm.24343 22711589

[34] Bilgic B, Chatnuntawech I, Fan AP, Adalsteinsson E. Regularized QSM in Seconds. In: Proc. Int. Soc. Mag. Reson. Med.; 2013 p. 0168.

[35] Zhao Y, Anderson AW, Gore JC. Computer simulation studies of the effects of dynamic shimming on susceptibility artifacts in EPI at high field. J Magnet Reson. 2005 3;173(1):10–22. Available from: http://www.ncbi.nlm.nih.gov/pubmed/15705507. 10.1016/j.jmr.2004.11.009 15705507

[36] Jackson JD. Classical Electrodynamics. Third edit ed John Wiley & Sons Inc; 1999.

[37] D’Errico J. polyfitn; 2006. Available from: http://www.mathworks.com/matlabcentral/fileexchange/34765-polyfitn.

[38] Smith SM. Fast robust automated brain extraction. Hum Brain Mapp. 2002 11;17(3):143–55. 10.1002/hbm.10062 12391568 PMC6871816

[39] Lindemeyer J, Oros-Peusquens AM, Vahedipour K, Shah NJ. Quality-based UnwRap of SUbdivided Large Arrays (URSULA) at 9.4T. In: Proc Intl Soc Magnet Reson Med. vol. 21; 2013 p. 2494.

